# First-episode psychiatric disorder risk from SARS-CoV-2 infection: A clinical analysis with Chinese psychiatric inpatients

**DOI:** 10.7555/JBR.38.20240005

**Published:** 2024-05-29

**Authors:** Ya Xie, Zifeng Xu, Yumin Zhang, Yisheng Li, Pengyu Du, Chun Wang

**Affiliations:** 1 Clinical Mental Health Center, the Affiliated Brain Hospital of Nanjing Medical University, Nanjing, Jiangsu 210029, China; 2 Medical School of Nanjing University, Nanjing, Jiangsu 210008, China

**Keywords:** COVID-19, SARS-CoV-2, psychiatric disorders, inflammation, interleukins

## Abstract

The extensive spread of severe acute respiratory syndrome coronavirus 2 (SARS-CoV-2) throughout China in late 2022 has underscored the correlation between this virus and severe psychiatric disorders. However, there remains a lack of reported clinical and pathological features. Accordingly, we retrospectively reviewed the electronic medical records of psychiatric inpatients for seven days from early January 2023. Twenty-one inpatients who developed first-episode psychiatric disorders within two weeks after SARS-CoV-2 infection were recruited, while 24 uninfected first-episode psychiatric inpatients were selected as controls. Comparative analyses of clinical manifestations, routine laboratory tests, and imaging examinations were performed. Our investigation demonstrated a 330% increase in the incidence of first-episode psychiatric inpatients after SARS-CoV-2 infection in 2023, compared with the preceding year without SARS-CoV-2 infections. Most cases exhibited psychiatric symptoms within one week of SARS-CoV-2 infection, which resolved after approximately two weeks, with no residual symptoms after three months. One-way ANOVA demonstrated a significant difference in the highest fever temperature between inpatients with and without psychotic symptoms. Infected inpatients displayed elevated levels of interleukin-4, interleukin-8, and interferon-α, but decreased levels of eosinophils and basophils. These findings suggest that SARS-CoV-2 may contribute to the development of psychiatric disorders, likely mediated by the virus-induced inflammatory response and neuronal dysfunction in the context of psychological distress.

## Introduction

On December 7, 2022, the Chinese government adjusted its epidemic prevention and control measures for coronavirus disease 2019 (COVID-19). Subsequently, the spread of the virus became rampant throughout China because of high population densities, leading to a cumulative rate of new coronavirus infections ranging from 60% to 80% in multiple provinces by the end of December. As the population recovered from the infection, we observed a significant increase in the first-episode of acute psychiatric disorders among patients in our psychiatric outpatient and inpatient wards, compared with the previous period. These patients exhibited severe psychiatric abnormalities after infection with severe acute respiratory syndrome coronavirus 2 (SARS-CoV-2), the causative strain of COVID-19, primarily manifesting as hallucinations, delusions, and disorganized thoughts, speech, and behavior^[1]^. Although no observable signs or symptoms of organic disease or direct causative factors were identified, the correlation did not seem coincidental. Varatharaj *et al*^[2]^ conducted a national interprofessional surveillance study on acute neurological and psychiatric complications of COVID-19, and found that 39 out of 125 investigated patients presented with psychiatric symptoms. A retrospective cohort study of COVID-19 cases estimated that the overall probability of diagnosing a new psychiatric disorder within 90 days of a confirmed COVID-19 diagnosis in 44759 patients without a history of psychosis was 5.8%^[3]^. Similar case reports have emerged, including a 36-year-old healthy female without a family history of psychiatric disorders who presented with significant persecutory delusions and decreased sleep four days after being diagnosed with COVID-19^[4]^.

SARS-CoV-2, a single-stranded RNA virus, exhibits a genetic structure and pathological effects similar to other coronaviruses previously prevalent in the population. This novel beta coronavirus shares 79% of its genome sequence with SARS-CoV and 50% with Middle East respiratory syndrome (MERS) coronavirus^[5–6]^. During the initial acute outbreaks of SARS and MERS, approximately 27%–41% of the cases experienced neuropsychiatric symptoms, with a few cases exhibiting psychosis and steroid-induced mania^[7]^. During the COVID-19 pandemic, a large-scale retrospective cohort study indicated that the risk of neurological and psychiatric diagnoses associated with COVID-19 was higher following the emergence of the Delta variant^[8]^. As a result, patients infected with SARS-CoV-2 may be at risk for certain psychiatric sequelae, according to past outbreaks and current reports of neuropsychiatric complications associated with COVID-19.

The brain is one of the target organs for coronaviruses, and it has been well-documented that β-coronaviruses, such as SARS-CoV-1, have neurotropism^[9–10]^. The detection of SARS-CoV-2 RNA and anti-SARS-CoV-2 antibodies in the cerebrospinal fluid of patients with acute encephalopathy caused by COVID-19 has been reported^[11–12]^. In brain autopsies performed on those patients who died of COVID-19, SARS-CoV-2 and pathological features associated with infection were also detected in cortical neurons^[13]^. Investigators have simulated the neuroinvasiveness of SARS-CoV-2 using human brain organoid and mouse models, and have observed a high replication potential of SARS-CoV-2 in the brain, which directly leads to massive neuronal death in human brain organoids^[14]^. These findings suggest that the psychiatric symptoms associated with SARS-CoV-2 infection may be related to direct viral invasion of the central nervous system (CNS), leading to a neurological damage.

Aside from a direct invasion of the CNS, SARS-CoV-2 also induces an increase in pro-inflammatory cytokines, especially interleukin (IL)-4, IL-6, IL-8, interferon (IFN), and tumor necrosis factor (TNF), triggering an immune response in the CNS and causing a subsequent neurological damage^[15–16]^. Studies have shown that pro-inflammatory cytokines exert effects on emotional behavior and cognition by diminishing levels of brain monoamines, activating neuroendocrine responses, promoting excitotoxicity (such as increasing glutamate levels), and impairing brain plasticity^[17]^. Psychiatric disorders, including mood disorders and schizophrenia, are known to be strongly associated with inflammatory factors^[18–19]^. Studies also found that anxiety and depressive symptoms in hospitalized patients with SARS-CoV-2 infection were associated with baseline inflammatory markers^[20]^. Moreover, the systemic inflammation index of patients was reported to predict the level of depression during the three-month follow-up period^[21]^. This so-called "cytokine storm" may be one potential mechanism for the development of psychiatric disorders in COVID-19 patients.

Although the risk of COVID-19 on psychiatric disorders has been proposed, there is a lack of reported clinical analyses addressing severe psychiatric disorders in psychiatric inpatients experiencing their first episode. Taking into account the unique social circumstances and widespread infection of SARS-CoV-2 in China, the current study investigated clinical manifestations, inflammatory markers, and neuroimaging of inpatients with the first-episode psychiatric disorders after SARS-CoV-2 infection, and examined the underlying pathobiological mechanisms.

## Subjects and methods

### Study design

The current study employed a case-control design based on a thorough screening of new admissions to the psychiatric department of Nanjing Brain Hospital between January 1 and January 7, 2023, which sees an average of 0.7 million outpatient visits annually. To identify eligible participants, a self-administered open-ended interview questionnaire was used, focusing on patients who met three specific inclusion criteria: (a) the first episode (without a prior psychiatric history); (b) confirmed SARS-CoV-2 infection within two weeks before the episode, as indicated by a positive antigen-antibody conjugated self-assay in nasal mucosal secretions or a positive fluorometric quantitative real-time reverse transcription polymerase chain reaction in throat swab specimens; and (c) no other severe stressful event stimuli or strong family history before the episode. Individuals exhibiting psychiatric symptoms because of somatic diseases or substance-induced conditions were excluded. Between January 1 and January 7, 2023, a total of 177 new inpatients were admitted to the psychiatric department, of which 24 were the first-episode patients with psychiatric disorders. Among the 24 patients, two were diagnosed with viral encephalitis one week after admission, and one had experienced a major stressful event before developing the disease.

To effectively mitigate confounding variables apart from SARS-CoV-2, a meticulous selection process was undertaken for the controls. The inclusion criteria outlined below were applied to the patients drawn from a randomized pool of 1000 psychiatric inpatients in 2022: (a) the first episode (without a prior psychiatric history); (b) a psychiatric disorder episode within two weeks before admission (both the infected group and the control group experienced acute episodes within two weeks); and (c) no prior history of SARS-CoV-2 infection. Patients with somatic diseases or substance-induced psychiatric disorders were excluded.

All clinical diagnoses and screenings were determined by at least two professional psychiatrists in a strict accordance with the International Classification of Diseases 10^th^ edition (ICD-10). Before including the cases in the analysis, an informed verbal consent was obtained from the patients or their legally authorized representatives in accordance with the principles of the Declaration of Helsinki. The current study has been approved by the Institutional Review Board of the Brain Hospital Affiliated to Nanjing Medical University (Ethics Approval No.: 2022/KY169/02 Fast), and the patients waived written informed consent.

### Data collection

Through individual interviews, pre-morbid personality, primary psychiatric diagnosis, typical symptoms, post-infection somatic symptoms, and prognosis of patients were collected from the infected group, and results of the chest computed tomography (CT) scans were queried to assess the extent of respiratory infection. In the comparison of pre-morbid personality traits between the two groups, we referred to Galen's temperament theory^[22]^ to categorize the pre-morbid personalities into five types for statistical analyses, including choleric, melancholic, sanguine, phlegmatic, and psychoticism. Any missing or uncertain information was addressed through a direct communication with their attending physicians and families. The case information, including age, sex, the length of hospitalization, the counts of peripheral blood cells (including counts of white blood cells, neutrophils, lymphocytes, mononuclear cells, eosinophils, and basophils), the peripheral plasma levels of C-reactive protein, and the results of 12 inflammatory factor tests, was organized by reviewing electronic medical records of the two groups of patients. All samples were collected and tested with enzyme-linked immunosorbent assay (ELISA) kits. Furthermore, 3.0T cranial magnetic resonance imaging (MRI) and sphenoid electrode electroencephalogram (EEG) were conducted to evaluate the neurological damage to exclude the possibility of organic disease.

### Statistical analysis

SPSS 26.0 was used for statistical analysis. The mean with standard deviation was used for normally distributed data, while the median with range was used for non-normally distributed data in descriptive analyses. Depending on the normality of the data, the two-sample Student's *t*-test and Mann-Whitney *U* test were selectively applied for further comparisons. The Chi-square test was used to compare groups by sex, and Fisher's exact test was used to compare groups by diagnosis, pre-morbid personality, and sphenoid electrode EEG. One-way ANOVA was used to examine the difference in febrile temperatures between patients with and without psychotic symptoms. The significance level was set at *P* < 0.05. Given that there was no pre-estimated sample size, we additionally calculated the *t* Cohen's *d* between the two-sample tests and performed power calculations in the G*power 3.1.94 program (https://www.gpower.hhu.de) for data with intergroup differences. The actual power was above 0.8, indicating an acceptable statistical efficacy of the difference between the groups.

## Results

### Clinical features and laboratory tests between the two groups

The demographic characteristics, diagnoses, and clinical psychiatric presentations of 21 hospitalized patients who developed their first psychiatric symptoms after SARS-CoV-2 infection are shown in ***Supplementary Table 1*** (available online). Among the randomly selected 1000 uninfected hospitalized patients in 2022, 24 patients with the first-episode psychiatric disorders who met the inclusion criteria were recruited as the control group.

There was no significant difference between the SARS-CoV-2-infected and the control groups in terms of age (*P* = 0.172) and sex (*P* = 0.739). The mean length of stay of infected patients was significantly shorter (*P* = 0.027) than that of the control group. The pre-morbid personality in the infected group was mostly choleric (33.3%) and melancholic (33.3%), while the control group was mostly melancholic (50.0%), but there was no significant difference in the pre-morbid personality traits between the two groups (*P* = 0.415) (***Table 1***).

**Table 1:** Comparisons of clinical characteristics, laboratory findings, and imaging results between the SARS-CoV-2-infected and control groups

Variables	Infected patients			Uninfected patients		*t/z/χ²*	Cohen's *d*	*P*
	*n*	mean±SD/ (median [range])		*n*	mean±SD/ [median (range)]			
Age [year, median (range)]	21	35.0 (16–75)		24	29.5 (16–50)	−1.366	0.417	0.172^a^
Sex (male/female, *n*)	21	8/13		24	8/16	0.111	–	0.739^b^
Length of stay [day, median (range)]	21	14 (7–44)		24	18.5 (10–94)	−2.214	0.675	0.027^a^
Diagnosis [*n* (%)]	21			24		17.165	–	<0.001^c*^
Acute psychotic disorder		3 (14.3)			16 (66.7)			
Manic episode with or without psychotic symptoms		7 (33.3)			7 (29.1)			
Depressive episode with or without psychotic symptoms		9 (42.9)			1 (4.2)			
Anxiety disorder		2 (9.5)			0 (0)			
Pre-morbid personality [*n* (%)]	21			24		4.135	–	0.415^c^
Choleric		7 (33.3)			3 (12.5)			
Melancholic		7 (33.3)			12 (50.0)			
Sanguine		4 (19.1)			5 (20.8)			
Phlegmatic		2 (9.5)			1 (4.2)			
Psychoticism		1 (4.8)			3 (12.5)			
Blood cell count (10^9^/L)	21			24				
White blood cells [median (range)]		7.05 (4.97–16.36)			5.93 (4.00–12.54)	−2.184	−0.666	0.029^a*^
Neutrophils (mean±SD)		66.72±11.92			58.53±11.42	2.354	0.702	0.023^d*^
Lymphocytes (mean±SD)		24.57±10.22			30.88±10.97	−1.987	−0.595	0.053^d^
Mononuclear cells [median (range)]		6.80 (3.50–14.60)			7.70 (5.10–10.80)	−1.229	−0.375	0.219^a^
Eosinophils [median (range)]		0.60 (0–7.10)			2.10 (0–6.2)	−3.906	−1.191	<0.001^a*^
Basophils [median (range)]		0.30 (0.10–0.60)			0.45 (0.20–0.80)	−2.352	−0.717	0.019^a*^
Inflammatory factor tests [pg/mL, median (range)]	19			24				
IL-2		3.15 (1.25–7.40)			2.01 (0.67–8.35)	−2.213	−0.691	0.027^a*^
IL-4		2.52 (1.32–5.21)			1.99 (0.90–4.44)	−2.656	−0.830	0.008^a*^
IL-5		3.44 (1.70–11.89)			2.67 (1.50–10.90)	−0.722	−0.830	0.471^a^
IL-6		2.28 (1.51–37.12)			3.62 (0.83–13.58)	−0.971	−0.303	0.332^a^
IL-8		25.67 (0.84–186.98)			4.78 (1.17–41.38)	−2.337	−0.730	0.019^a*^
IL-10		1.89 (1.10–7.36)			1.68 (1.03–4.29)	−1.162	−0.363	0.245^a^
IL-17		2.11 (2.11–43.04)			4.36 (1.05–67.55)	−1.842	−0.575	0.065^a^
IL-1β		2.77 (1.77–54.91)			2.76 (0.67–32.79)	−0.049	−0.015	0.961^a^
IL-12p70		1.79 (1.43–3.74)			1.98 (1.09–4.63)	−1.042	−0.325	0.298^a^
IFN-α		3.43 (1.22–13.53)			1.63 (0.87–6.56)	−2.813	0.879	0.005^a*^
IFN-γ		4.54 (1.95–11.19)			3.86 (1.49–15.73)	−1.039	−0.325	0.299^a^
TNF		2.64 (1.17–39.97)			2.20 (1.17–31.91)	−0.881	−0.275	0.378^a^
C-reactive protein [ml/L, median (range)]	16	<5.00 (<5.00–35.71)^e^		24	<5.00 (<5.00–6.72)^e^			

The laboratory findings demonstrated that the infected patients had significantly elevated counts of white blood cells (*P* = 0.029) and neutrophils (*P* = 0.023), but reduced counts of eosinophils (*P* < 0.001) and basophils (*P* = 0.019), compared with the control group. In terms of inflammatory factors, the peripheral blood of the infected individuals exhibited increased expression levels of cytokines IL-2 (*P* = 0.027), IL-4 (*P* = 0.008), IL-8 (*P* = 0.019), and IFN-α (*P* = 0.005) compared with those of the control group. We further performed a power calculation for the inflammatory factors that differed between the infected and the control groups, and found that the actual power of eosinophils, basophils, IL-4, IL-8, and IFN ranged from 0.818 to 0.998, indicating a sufficient statistical efficacy (***Table 1***).

Additionally, abnormal waves detected by EEGs were observed in 38.9% of patients from the infected group. Cranial MRI scans further revealed the presence of several cases of paranasal sinusitis, frontal arachnoid cysts, and abnormal signals in the frontoparietal subcortex. Similarly, the chest CT scans revealed the possibility of lung inflammation in two of the patients, while most patients presented with mild symptoms and did not develop pneumonia (***Table 1***).

### SARS-CoV-2 and psychiatric symptoms

**Figure 1:**
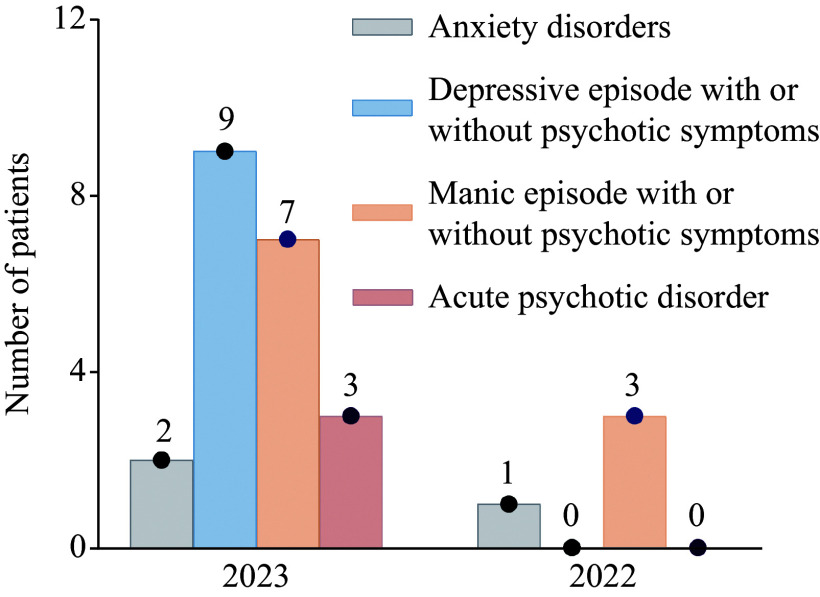
The number of hospitalized patients with the first-episode psychiatric disorders admitted from January 1 to 7 in 2023 and 2022.

Compared with January 1–7, 2022 (when no inpatients were infected with SARS-CoV-2), only four of 145 new admissions to the psychiatric departments experienced their first psychiatric symptoms within two weeks; the incidence of inpatients with the first-episode psychiatric disorders after the infection at the beginning of 2023 increased by 330%, compared with that in the same period of the previous year without infection (***Fig. 1***). The average age of the aforementioned 21 patients was 37.86 years, eight of whom were male. There were three cases of acute psychotic disorder (14.3%), seven cases of manic episode with or without psychotic symptoms (33.3%), nine cases of major depressive episode with or without psychotic symptoms (42.9%), two cases of anxiety state and panic episodes (9.5%), with one patient was diagnosed with schizophrenia after a month (***Fig. 1***). Fifteen of these patients exhibited a short interval of one week between the infection and the episode of psychiatric symptoms, while the remaining six patients showed a relatively longer interval within two weeks. All the participants included in the current study had not used steroid drugs after infection, 15 of whom had used non-steroidal anti-inflammatory drugs for fever reduction. For the treatment of psychiatric symptoms, only common antipsychotics, antidepressants, and benzodiazepines were selected. One month after discharge, eight of the patients continued to suffer from anxiety and emotional distress, but only one person was readmitted with disturbed speech and behavior, while the remaining patients became asymptomatic. None of the patients had any substantial residual symptoms after the three-month follow-up (***Table 2***).

**Table 1:** Comparisons of clinical characteristics, laboratory findings, and imaging results between the SARS-CoV-2-infected and control groups (continued)

Variables	Infected patients			Uninfected patients		*t/z/χ²*	Cohen's *d*	*P*
	*n*	mean±SD/ [median (range)]		*n*	mean±SD/ [median (range)]			
Sphenoid electrode EEG [*n* (%)]	18			11		3.665	–	0.182^c^
Normality		11(61.1)			9 (81.8)			
Mild abnormality		5 (27.8)			0 (0)			
Moderate-severe abnormality		2 (11.1)			2 (18.1)			
Cranial MRI [*n* (%)]	19			24		2.516	–	0.113^b^
Normality		14 (73.7)			22 (91.7)			
Abnormality		5 (26.3)			2 (8.3)			
Chest CT scan [*n* (%)]	21^f^			–				
Normality		10 (47.6)						
Inflammation		2 (9.5)						
Bronchial vascular shadows		4 (19.0)						
Pleural thickening		3 (14.3)						
Cord-like shadow		2 (9.5)						
Others		2 (9.5)						
^a^Tested by Mann-Whitney *U*-test.^b^Tested by Chi-square test.^c^Tested by Fisher's exact test.^d^Tested by a two-sample Student's *t*-test.^e^C-reactive protein less than 5 ml/L does not show specific values when measured.^f^Each patient might have more than one lung manifestation.^*^Power calculations were performed on the *P*-values. The actual power was 0.767 for white blood cells, 0.632 for neutrophils, 0.998 for eosinophils, 0.825 for basophils, 0.774 for IL-2, 0.903 for IL-4, 0.818 for IL-8, and 0.932 for IFN-α. The actual power was above 0.8 for eosinophils, basophils, IL-4, and IL-8, indicating that there was a greater statistical power for the difference in inflammatory factors between the infected and control groups.Abbreviations: SD, standard deviation; IL, interleukin; IFN, interferon; TNF, tumor necrosis factor.								

**Table 2:** Clinical characteristics of patients after the SARS-CoV-2 infection

Clinical characteristics	Infected patients (*n*=21)
Age (year, mean±SD)	35 (16–75)
Sex (male/female, *n*)	8/13
Interval between infection and episode [*n* (%)]	
Within one week	15 (71.4)
Within two weeks	6 (28.6)
Prognosis (residual symptoms) [*n* (%)]	
One-month follow-up	9 (42.9)
Three-month follow-up	0 (0)
Post-infection symptoms [*n* (%)]	
Fever	19 (90.5)
Cough and sore throat	14 (66.7)
Smell and taste impairment	3 (14.3)
Loss of appetite	6 (28.6)
Muscle soreness	6 (28.6)
Fatigue	10 (47.1)
Palpitation and chest tightness	5 (23.8)
Dizziness and headache	9 (42.9)
Insomnia	17 (81.0)
Attention and memory decline	5 (23.8)
Abbreviation: SD, standard deviation.	

### SARS-CoV-2 and psychotic symptoms

**Figure 2:**
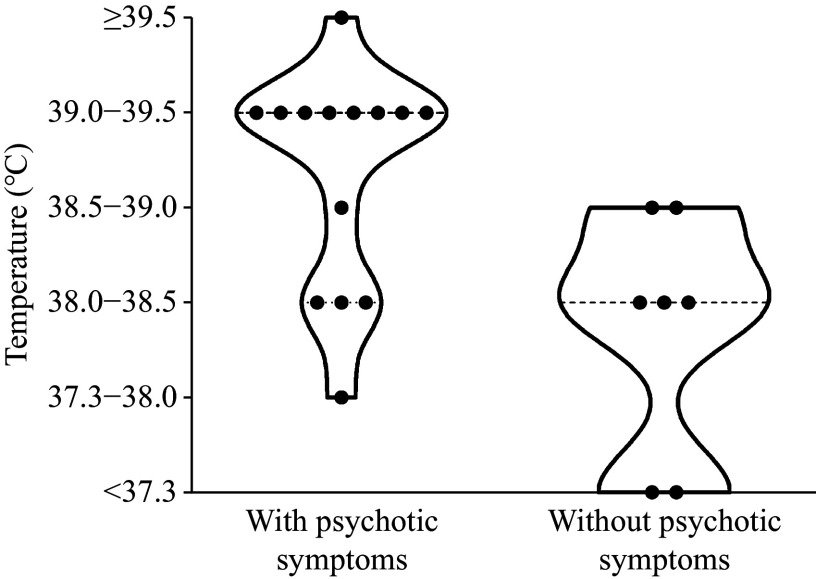
Fever temperature distributions in the infected patients with or without psychotic symptoms. The highest temperature of fever in infected patients was recorded after the SARS-CoV-2 infection and before the first episode of psychiatric symptoms.

Of the 21 inpatients who developed first-episode psychiatric disorders within two weeks after SARS-CoV-2 infection, 14 demonstrated psychotic symptoms characterized by hallucinations, thought disorders, catatonia, disorganized speech and behavior, and other schizophrenia-like symptoms. Nine patients presenting with psychotic symptoms demonstrated a peak temperature exceeding 39 ℃, whereas those infected patients without psychotic symptoms presented a maximum temperature below 39 ℃ (***Fig. 2***). A significant between-group effect was observed through one-way ANOVA, with patients exhibiting psychotic symptoms showing significantly higher temperature scores than those without psychotic symptoms (*F* = 3.462, *P* = 0.028, ***Table 3***). This finding suggests a correlation between the presence of psychotic symptoms and maximum fever temperature. Besides fever, the most prevailing somatic manifestations of these patients after infection were insomnia [17 (81.0%)], followed by cough and sore throat [14 (66.7%)], fatigue [10 (47.6%)], dizziness and headache [9 (42.9%)], with a small proportion of patients suffering from loss of appetite [6 (28.6%)], muscle soreness [6 (28.6%)], palpitations and chest tightness [5 (23.8%)], attention and memory decline [5 (23.8%)], and smell and taste impairment [3 (14.3%)] (***Table 2***).

**Table 3:** One-way ANOVA of the highest fever temperature of the SARS-CoV-2-infected patients with and without psychotic symptoms

Variation	*n*	Temperature score^a^ (mean±SD)	MS	*F*	*P*
Presence with psychotic symptoms	14	4.36±1.15	0.500	3.462	0.028
Presence without psychotic symptoms	7	2.71±1.25			
^a^The highest fever temperature of the infected patients was classified with a score range between one and six as follows: 1 (<37.3 ℃); 2 (37.3–38.0 ℃); 3 (38.0–38.5 ℃); 4 (38.5–39.0 ℃); 5 (39.0–39.5 ℃); 6 (≥ 39.5 ℃). Abbreviations: SD, standard deviation; MS, mean square.					

## Discussion

The current study revealed a noteworthy rise in the percentage of first-episode psychiatric hospitalizations after SARS-CoV-2 infection, implicating a potential predisposing effect of SARS-CoV-2 on psychiatric disorders.

Most infected inpatients displayed psychiatric disturbances within a week of infection, which subsided in approximately two weeks. Their chest CT results after admission also suggested that the pulmonary inflammation was gradually regressing, indicating that the remission of inflammation yields substantial relief from the associated symptoms. It has been demonstrated that the intensity of inflammatory markers is associated with the fluctuation of clinical symptoms in psychiatric disorders^[23]^. Notably, our ANOVA on the highest fever temperature and delusion diagnosis suggested that the psychotic symptoms might be associated with a higher viral load resulting from SARS-CoV-2 infection^[24]^. The COVID-19 patients demonstrated elevated concentrations of ILs and IFNs, which implied an upregulation in the function of T-helper 1 cells, leading to heightened secretion of cytokines^[25]^. Serum concentrations of pro-inflammatory factors, such as IFN-α, IL-6, and IL-8, may serve as potential markers of psychiatric disorders, as inflammation in the CNS is associated with neurodegeneration^[26]^. Both IL-4 and IL-8 have demonstrated pleiotropic effects in terms of pro- and anti-inflammatory aspects^[27]^. IL-8 levels have tended to be elevated in several psychiatric disorders and appeared to be the most sensitive marker in cerebrospinal fluid^[28]^. A clinical study showed that the elevated maternal levels of IL-8 increased the likelihood of schizophrenia in adult offspring^[29]^. Likewise, adolescents experiencing their initial episode of schizophrenia exhibited a strong correlation between negative symptoms and the elevated levels of IL-4^[30]^. The IFN-α signaling represents a major target for regulating viral latency action^[31]^. The virus-host interaction hypothesis for SARS-CoV-2 has presented some evidence for IFN-α dysregulation. Currently, its consequential neurocognitive disorder has been explored in association with this dysregulated pathway^[32]^. Alternatively, IFN has been postulated as a biomarker for the post-COVID depression^[33]^. Studies observed a negative correlation of basophils and eosinophils with the manifestation of anxiety and somatization symptoms as indicated by the scores on the Hamilton Depression Scale, and the decreased basophils specifically appear to play a prominent role in the manifestation of anxious depression^[34]^. This may be correlated with the stimulation of cortisol secretion and the subsequent induction of apoptosis under the stress of a new coronavirus infection^[35]^. The decrease in basophils affects histamine release, which in turn leads to increased anxiety levels in individuals^[36]^.

Taken together, these findings further highlight the potential for pro- and anti-inflammatory states to influence the pathological manifestations of psychiatric disorders. SARS-CoV-2 may induce a cytokine storm and lead to autoimmune dysregulation. When SARS-CoV-2 replicates in the host, the immune response is activated, mediated by membrane-bound immune receptor subtypes and downstream signaling pathways^[37]^. This response involves a massive infiltration of monocytes and macrophages that secrete cytokines, chemokines, and other inflammatory mediators contributing to the cytokine storm^[38–39]^. Meta-analyses have demonstrated an increase in blood cytokine levels in patients with the first-episode psychosis and acute exacerbation of schizophrenia, including IL-6, IL-17, IL-8, IFN-γ, and TGF-β^[18]^. Additionally, the levels of IL-6, IL-8, TNF-α, and IL-4 were found to increase during the acute manic phase of bipolar disorder^[19]^. The cells of microglia and astroglia within the CNS play an integral role in the regulation of immune inflammation by responding to both pro- and anti-inflammatory cytokine signals^[15]^. Even if the virus fails to penetrate directly into the CNS, peripheral cytokines involved in the host's antiviral response may provoke an inflammatory response that increases the permeability of the blood-brain barrier, allowing peripheral immune cells to pass into the CNS and disrupt neurotransmission, thereby inducing psychiatric symptoms^[40]^.

As observed in the reported physical examination findings, the COVID-19 patients may experience an elevated fronto-parietal white matter high signal volume, abnormal frontotemporal discharge, and substantial structural damage^[41–42]^. These alterations in frontotemporal microstructural and functional connectivity are known to be associated with a heightened risk of developing a psychotic disorder^[43]^. Imaging studies have shown that individuals after the SARS-CoV-2 infection exhibit diffuse white matter lesions that are believed to be associated with oligodendrocyte death and subsequent acute demyelination^[44]^. Upon binding to angiotensin-converting enzyme 2 (ACE2) receptors in the capillary endothelium, the SARS-CoV-2 stinger protein may disrupt the blood-brain barrier and enable the virus to invade the CNS^[45]^. The astrocytes and oligodendrocyte precursor cells, which are widely present in periventricular organs, contain 1.9% and 1.6% ACE2-expressing cells, respectively^[46]^. The invasion of the CNS by SARS-CoV-2 may lead to demyelination and neurodegeneration, as well as a pro-inflammatory response, subsequently inducing psychiatric symptoms^[47]^.

In the current investigation, patients with melancholic temperament often exhibited anxiety and concerns about the SARS-CoV-2 infection, suggesting that COVID-19 may increase mental stress in susceptible individuals. During the pandemic, susceptible individuals were reported to have been exposed to multiple stressors, such as fear of infection and recurrence, nervousness about somatic conditions after infection, and concerns about the health status of family members of susceptible individuals, further exacerbating the predisposition to mental illness under inflammatory stimuli^[20,48]^. ACE2 is widely expressed across various organ tissues and is essential for the development of psychiatric symptoms after SARS-CoV-2 infection^[14,46]^. The binding of SARS-CoV-2 to the ACE2 receptors may lead to the downregulation of ACE2 expression, which may enhance sympathetic activity and trigger the inflammatory response, resulting in reduced 5-hydroxytryptamine levels^[49]^. Meanwhile, chronic mental stress may lead to dysregulation of the hypothalamic-pituitary-adrenal stress axis feedback mechanism, causing abnormal corticosteroid secretion in the body, which promotes a sustained oxidative stress response^[50]^. Studies have demonstrated the effects of a sustained and unpredictable stress on monoamine and oxidative levels in the frontal cortex, striatum, and hippocampus of rats^[51]^, with inflammation and oxidative stress being significant contributors to depression and anxiety progression^[52]^.

The sequelae of COVID-19, especially dizziness and headache, fatigue, insomnia, and cognitive impairment, were also observed and raised more concern. A comprehensive meta-analysis, using a multifactorial approach, reported that approximately 80% of the infected patients might develop at least one long-term symptom by a multifactorial mechanism^[53–54]^, and of all the symptoms associated with depression and anxiety, fatigue, and insomnia were most tightly associated with inflammatory markers^[55]^. Some of these symptoms may be attributed to the direct infection by SARS-CoV-2, because when it enters the olfactory support cells, it causes smell loss. Other symptoms, such as dizziness and headache, may be associated with the hypercoagulable state of cerebral vessels arising from inflammation^[56]^. The cognitive decline may be associated with chronic inflammation and microstructural changes in the CNS, as well as aspects of tissue hypoxia, autoimmunity, and endocrine metabolic disorders^[57–58]^. Studies have found that the viral infection induces localized tissue hypoxia and further reduces the tissue damage threshold^[14]^.

In our case, as well as in some other reports, atypical antipsychotics, selective 5-hexamine re-intake inhibitors, and benzodiazepines are essential components for the therapy of psychiatric symptoms, and a modified electroconvulsive therapy is an option, if the medication is ineffective^[4,59]^. Given the pronounced inflammatory response associated with this psychiatric disorder, antipsychotics with anti-inflammatory may be preferred^[32]^. Regarding the treatment of long-term sequelae, appropriate exercise may enhance the expression of ACE2, thereby improving mental and physical health^[60]^. The current study revealed that essentially all the patients had no residual symptoms after three months, which is consistent with several available case reports^[61–62]^, suggesting a positive prognosis for this psychiatric disorder.

However, there are some limitations to the current study. The sample size was relatively small, and although we endeavored to exclude confounding factors, such as stressful events, physical illnesses, and family history, there might still be some potential biases affecting the results. Secondly, this investigation was limited to the inpatient psychiatric ward in China, and it does not reflect the mental status of the overall Chinese populace or outpatients after infection. Therefore, although the current study revealed an association between COVID-19-mediated inflammatory responses and the first-episode psychiatric disorders, future large-scale investigations with multiple clinical centers are needed to verify the accuracy and reliability of these findings.

In summary, our findings indicate an increased risk of the first-episode psychiatric disorders associated with the COVID-19 pandemic. The increased levels of white blood cell counts and high expression levels of cytokines in response to the SARS-CoV-2 infection may play a crucial role in the pathogenesis of the disease in psychiatric patients. Although the precise neurobiological mechanism of how SARS-CoV-2 contributes to psychiatric symptoms remains to be elucidated, inflammatory infiltration and neuronal damage in the CNS under the stress of COVID-19 could be the primary causes of psychosis. Furthermore, large-scale and even prospective epidemiological studies are required to clarify the susceptibility to psychiatric symptoms and different symptom expressions after SARS-CoV-2 infection.

## SUPPLEMENTARY DATA

Supplementary data to this article can be found online.
