# Supplementary material for: First-episode psychiatric disorder risk from SARS-CoV-2 infection: A clinical analysis with Chinese psychiatric inpatients
Source: J Biomed Res. 2024 May 29;39(1):50–60. doi: 10.7555/JBR.38.20240005 (PMC11873594; doi:10.7555/JBR.38.20240005)
Supplement: Supplementary file 1 — Supplementary data to this article can be found online. [file jbr-39-1-50-S1.pdf]

Available online at www.jbr-pub.org.cn

Open Access at PubMed Central

Journal of Biomedical Research, 2025 39(1): 1–2

Supplementary Data

JBR

First-episode psychiatric disorder risk from SARS-CoV-2 infection: A clinical analysis with Chinese psychiatric inpatients

Ya Xie[1,△], Zifeng Xu[1,△], Yumin Zhang[1], Yisheng Li[1], Pengyu Du[2], Chun Wang[1,✉]

[1]*Clinical Mental Health Center, the Affiliated Brain Hospital of Nanjing Medical University, Nanjing, Jiangsu 210029, China;*
[2]*Medical School of Nanjing University, Nanjing, Jiangsu 210008, China.*

Supplementary Table 1 **Summary of demographic characteristics, diagnosis, and clinical psychiatric presentation of the SARS-CoV-2 infection inpatients**

Patient ID	Age (year)	Sex	Family psychiatric history	Pre-morbid personality	Psychiatric diagnosis	Psychiatric presentation
No. 1	35	F	None	Introverted	Acute and transient psychotic disorder	Disordered speech and behavior, delusions of being monitored and threatened by her in-laws
No. 2	42	F	None	Sentimental, inferior, and timid	Major depressive episode with psychotic symptoms	A sub-catatonic of passive negativism, non-speaking and non-responsive behavior, refusal to eat, alert and hostile
No. 3	23	F	None	Impatient and capricious, lacking self-control	Manic episode	Extensive shopping and spending, exaggerated delusions of her abilities, hyper-energy, and frequent conflicts with others
No. 4	51	F	None	Mild and introverted	Major depressive episode with psychotic symptoms	Disordered speech and behavior, childishness, nervousness, and fear
No. 5	16	F	None	Optimistic and outgoing, impatient and irritable	Major depressive episode with psychotic symptoms	Retardation of thinking, depression, babbling, frequent dry heaving, fear of the police, and fragmentary delusions
No. 6	27	M	None	Mild and calm, sentimental	Manic episode	Excessive talking, shouting, staying awake all night, exaggerated delusions such as predicting the future
No. 7	19	F	None	Enthusiastic and motivated	Major manic episode with psychotic symptoms	Exaggerated delusions regarding her status and finances, bizarre behavior, self-talk, and laughter
No. 8	16	M	None	Suspicious and sensitive	Manic episode	Exaggeration of self-competency, flight of thought, impulsive destruction of objects, fragmentary delusions of persecution
No. 9	46	F	None	Optimistic and outgoing	Manic episode	Exaggerated delusion of self-competency, flight of thought that talking endlessly, sexual exuberance, a belief that mother is a trafficker
No. 10	30	M	None	Impatient and irritable	Manic episode	Delusions of identity with religious themes, a belief in the ability to cure COVID-19, decreased need for sleep
No. 11	17	M	None	Impatient	Manic episode	Exaggerated delusion of self-power, gambling

△These authors contributed equally to this work.

✉Corresponding author: Chun Wang, the Affiliated Brain Hospital of Nanjing Medical University, 264 Guangzhou Road, Nanjing, Jiangsu 210029, China. E-mail: chun_wang@njmu.edu.cn.

Received: 07 January 2024; Revised: 27 March 2024; Accepted: 07 April 2024; Published online: 29 May 2024

CLC number: R749, Document code: A

The authors reported no conflict of interests.

This is an open access article under the Creative Commons Attribution (CC BY 4.0) license, which permits others to distribute, remix, adapt and build upon this work, for commercial use, provided the original work is properly cited.

© 2025 by Journal of Biomedical Research.

https://doi.org/10.7555/JBR.38.20240005

Supplementary Table 1 **Summary of demographic characteristics, diagnosis, and clinical psychiatric presentation of the SARS-CoV-2 infection inpatients** (continued)

Patient ID	Age (year)	Sex	Family psychiatric history	Pre-morbid personality	Psychiatric diagnosis	Psychiatric presentation
No.12	32	M	None	Optimistic and outgoing	Anxiety state	Insomnia and weakness, convincing he has developed depression, and feeling terrified and hopeless
No.13	26	F	History of psychosis in grandmother	Optimistic and outgoing	Major depressive episode without psychotic symptoms	A fear of having serious complications, emotional breakdown, crying, nervousness, eating or sleeping disorders
No.14	75	M	None	Optimistic and outgoing, impatient and irritable	Depressive episode	Insomnia and dreaminess, fatigue and tiredness, low emotion
No.15	53	M	None	Impatient and irritable	Panic episode	Sudden onset of dyspnea, tremors, and tightness in the limbs while watching TV, which lasts for 20 minutes and then relieves itself
No.16	66	F	None	Optimistic and outgoing	Depressive episode	Overly worried about the health of the family after the infection, insomnia, chest tightness, and a belief in her limited lifespan
No.17	35	F	None	Careful and assertive	Depressive episode	Fear of deterioration of physical condition after infection, followed by depressed mood, loss of interest, and hopelessness
No.18	36	F	None	Introverted, sentimentally	Schizophreniform psychosis	Rambling about death, impulsive and irritable, behaving weirdly
No.19	27	M	Grandfather had experienced depression	Introverted	Schizophrenia	Babbling about the apocalyptic rhetoric and ghosts, stripping naked and running outside, a delusion of persecution
No.20	65	F	None	Optimistic, moderation and patience	Depressive episode	Insomnia, depressed mood, concerning about the news of the epidemic, fearing of potential complications
No.21	58	F	None	Introverted	Depressive episode	Insomnia, low emotion, fatigue, loss of energy and appetite, and thoughts of death

Abbreviations: F, female; M, male.
